# Photosynthetic Protein-Based Edible Quality Formation in Various *Porphyra dentata* Harvests Determined by Label-Free Proteomics Analysis

**DOI:** 10.3390/cells11071136

**Published:** 2022-03-28

**Authors:** Mingchang Yang, Lizhen Ma, Xianqing Yang, Laihao Li, Shengjun Chen, Bo Qi, Yueqi Wang, Chunsheng Li, Ya Wei, Yongqiang Zhao

**Affiliations:** 1 Key Laboratory of Aquatic Product Processing, Ministry of Agriculture and Rural Affairs, National R&D Center for Aquatic Product Processing, South China Sea Fisheries Research Institute, Chinese Academy of Fishery Sciences, Guangzhou 510300, China; 2005028144@stu.tjau.edu.cn (M.Y.); yangxq@scsfri.ac.cn (X.Y.); lilaihao@scsfri.ac.cn (L.L.); chenshengjun@scsfri.ac.cn (S.C.); qibogd@scsfri.ac.cn (B.Q.); wangyueqi@scsfri.ac.cn (Y.W.); lichunsheng@scsfri.ac.cn (C.L.); weiya@scsfri.ac.cn (Y.W.); 2 College of Food Science and Bioengineering, Tianjin Agricultural University, Tianjin 300384, China; mali@tjau.edu.cn; 3 Co-Innovation Center of Jiangsu Marine Bio-Industry Technology, Jiangsu Ocean University, Lianyungang 222005, China; 4 Collaborative Innovation Center of Seafood Deep Processing, Dalian Polytechnic University, Dalian 116034, China

**Keywords:** different harvest, formation mechanism, photosynthetic, *Porphyra dentata*, proteomics

## Abstract

The influence of harvest time on the photosynthetic protein quality of the red alga *Porphyra dentata* was determined using label-free proteomics. Of 2716 differentially abundant proteins that were identified in this study, 478 were upregulated and 374 were downregulated. The top enriched Kyoto Encyclopedia of Genes and Genomes (KEGG) and gene ontology (GO) pathways were metabolic processes and biosynthetic pathways such as photosynthesis, light harvesting, and carbon fixation in photosynthetic organisms. Nine important photosynthetic proteins were screened. Correlations among their expression levels were contrasted and verified by western blotting. PSII D1 and 44-kDa protein levels increased with later harvest time and increased light exposure. Specific photoprotective protein expression accelerated *P. dentata* growth and development. Biological processes such as photosynthesis and carbon cycling increased carbohydrate metabolism and decreased the total protein content. The results of the present study provide a scientific basis for the optimization of the culture and harvest of *P. dentata*.

## 1. Introduction

*Porphyra dentata* (*P. dentata*) is an edible red seaweed belonging to the Phylum Rhodophyta (Class Protoflorideae). In recent years, it has been widely cultivated, harvested, processed, and consumed in East-Asian countries. Southern China extensively cultivates and produces seaweeds [1,2,3]. As consumer health awareness increases, the demand for *Porphyra* in food processing is increasing rapidly. *Porphyra* is rich in proteins, carbohydrates, vitamins, micronutrients, and essential amino acids [4,5]. It is also abundant in vitamin B12, porphyrans, and taurine [6,7,8]. *P. dentata* has significantly higher mineral and amino acid content than other *Porphyra* species [9]. Certain constituents of *P. dentata* are bioactive and may have antineoplastic and other pharmacological efficacy. This alga is also rich in polysaccharides and is used to prepare various peptide products and tea beverages [10,11,12].

The *P. dentata* harvest period is from November to March. It is cultivated by the split stubble method, and it is harvested four to five times annually. The harvests are designated the first to the fourth or fifth harvests [13,14]. The protein levels from the first and fifth harvest are approximately 35% and 31%, respectively. Meanwhile, the carbohydrate levels in the first and fifth harvest are 29% and 31%, respectively (Yang M, Zhao Y, Wang J, et al., unpublished data). The nutritive value gradually decreased with harvest time.

In addition to changes due to longer harvest times, different processing methods had a considerable impact on *P. dentata* nutrient composition and content. The thalli of the first harvest are crisp, have a high protein content, and are rich in bioactive ingredients. They may be eaten raw or processed into various products such as sun-dried, baked, or sliced laver and salads. The thalli of the fifth harvest are thick, black, and cheap. They are used as food additives or non-food biological materials. Hence, the harvest period of *P. dentata* determines the industrial application of this alga.

Sunlight provides the energy required for the growth of *P. dentata* and affects its temperature and light stress responses [15]. *P. dentata* growth, development, and physiology are all influenced by ambient temperature, ultraviolet (UV) irradiation, and light wavelength and intensity. A previous study showed that the total soluble protein (TSP) content decreased while the growth rate increased with increasing light intensity and sunlight duration [16]. The relative photosynthetic response of the alga varies with regional climate and aquatic physicochemistry [17]. Observed differences in *P. dentata* photosynthesis are associated with biotic and abiotic stressors, including temperature extremes, ocean acidification, carbon fixation rates, tides, salinity level [18,19,20].

Recently, proteomics has been widely used to evaluate red algal edible quality. For example, these methods revealed that *Porphyra haitanensis* counteracts drought stress mainly by downregulating its own photosynthetic and energy metabolism activity. They were also used to identify the antioxidant enzymes involved in photosynthesis and protein synthesis in wild-type *Porphyra yezoensis* as well as irradiation-induced mutants [21,22] Label-free proteomics operates stably and can significantly reveal detailed information about the changes in proteins that occur in response to different conditions. It can also identify potential biomarkers [23]. To the best of our knowledge, the present study was the first to apply proteomics to *P. dentata* during different harvest periods and assess differential expression of the proteins related to the observed variations in quality between first-harvest and fifth-harvest *P. dentata*. The factors implicated in algal harvest quality formation were investigated from the perspective of photosynthesis. The objectives of this study included the provision of a theoretical basis for quantifying the quality grading standards of *P. dentata* and a scientific basis for its effective development and utilization at different harvest times.

## 2. Materials and Methods

### 2.1. Samples

First- and fifth-harvest *P. dentata* were obtained from Shen’ao Bay (23.46° N, 117.09° E), Nan’ao Island, Shantou City, Guangdong Province, China, in December 2020. They were cleaned with sterile water to remove sediment, appressoria, and other impurities, placed in sterile sealed tubes, transported to the laboratory in liquid nitrogen, and stored at −80 °C.

### 2.2. Total Protein Extraction

The samples were ground in liquid nitrogen and BPP (borax, polyvinylpolypyrrolidone [PVPP], and phenol) mixed at a 1:10 ratio. The suspensions were centrifuged at 12,000× *g* and 4 °C for 20 min, and the supernatants were collected. An equal volume of Tris-saturated phenol was added to each supernatant, and each mixture was vortexed at 4 °C for 10 min. The mixtures were centrifuged (3K30, Sigma, St. Louis, MO, USA) at 12,000× *g* and 4 °C for 20 min, and the phenol phases were collected. An equal volume of BPP was added to each supernatant and the mixtures were vortexed at 4 °C for 10 min. The solutions were centrifuged at 12,000× *g* and 4 °C for 20 min, and the phenol phases were collected. Five volumes pre-cooled 0.1 M onium acetate in methanol were added, and the proteins were precipitated at −20 °C overnight. The supernatants were discarded by centrifugation (12,000× *g*), and the precipitates were washed twice with 90% (*v*/*v*) acetone and then air-dried. The precipitates were resuspended in lysis buffer (1% (*v*/*v*) SDS plus 8 M urea) and sonicated on ice for 3 min. The lysates were centrifuged and the supernatants were collected to determine the protein content by the bicinchoninic acid (BCA) method in a BCA Protein Assay Kit (Beyotime Biotechnology, Shanghai, China). The proteins were quantified according to the kit protocol.

### 2.3. Protein Digestion

Protein digestion was performed according to a standard procedure [24]. Each sample tube contained 100 μg protein, and Tris (2-carboxyethyl) phosphine (TCEP) was added to a final concentration of 10 M. The tubes were incubated at 37 °C for 60 min. Appropriate amounts of iodoacetamide (IAM) were added to a final concentration of 40 M and reacted in the dark for 40 min. Then six volumes of cold acetone were added to each sample tube. The tubes were inverted three times and incubated at −20 °C for 4 h or until precipitates formed. The acetone was removed by centrifugation at 10,000× *g* for 20 min, and the precipitated protein was resuspended in 150 µL of 100 M triethylammonium bicarbonate (TEAB) buffer. Then 1:50 trypsin solution was added to each sample tube, and the mixtures were incubated at 37 °C overnight.

### 2.4. Peptide Desalination and Quantification

The peptides were vacuum-dried (LNG—T83, Huamei, Jiangsu, China) and resuspended in 2% (*v*/*v*) acetonitrile and 0.1% (*v*/*v*) trifluoroacetic acid (TF). The samples were desalted with Sep-Pak (Waters Corp., Milford, MA, USA) and vacuum-dried. The peptide concentrations were determined with a Peptide Quantification Kit (No. 23275; Thermo Fisher Scientific, Waltham, MA, USA). Loading buffer was added to each tube to prepare the samples for mass spectrometry (MS) analysis. The concentration of each sample was 0.5 µg/µL.

### 2.5. Liquid Chromatography-Tandem Mass Spectrometry (LC–MS/MS) Analysis

The peptides were dissolved in mass spectrometry loading buffer and analyzed by LC–MS/MS. The peptide samples were separated in an EASY-nLC 1200 Liquid System (Thermo Fisher Scientific). A C18 chromatographic column was used (75 μm × 25 cm; Thermo Fisher Scientific). Mobile phase A was 2% (*v*/*v*) acetonitrile plus 0.1% (*v*/*v*) formic acid. Mobile phase B was 80% (*v*/*v*) acetonitrile plus 0.1% (*v*/*v*) formic acid. The separation gradient was (1) 0–2 min: mobile phase B linearly increased from 0 to 6%; (2) 2–105 min: mobile phase B linearly increased from 6% to 23%; (3) 105–130 min: mobile phase B linearly increased from 23% to 29%; (4) 130–147 min: mobile phase B linearly increased from 29% to 38%; (5) 147–148 min: mobile phase B linearly increased from 38% to 48%; (6) 148–149 min: mobile phase B linearly increased from 48% to 100%; (7) 149–155 min: mobile phase B held at 100%. Mass spectrometry was conducted in Q-Exactive HF-X (Thermo Fisher Scientific). The MS scanning range (*m*/*z*) was 350–1300, the acquisition mode was DDA, the fragmentation mode was HCD, the primary MS resolution was 70,000, and the secondary MS resolution was 17,500.

### 2.6. Protein Identification

The original MS data output files were imported into the Proteome Discoverer^TM^ v. 2.2 software System (Thermo Fisher Scientific) for database searching and analysis. The fixed modification was carbamidomethyl, the variable modification was oxidation, the digested protein was trypsin, the maximum allowable error range of the parent ion mass was ±10 ppm, and the false discovery rate (FDR) of the peptide identification was ≤0.01.

The identified protein contained at least one specific peptide. The protein abundance information obtained from the database search was used to perform statistical tests and analyses on the differentially abundant proteins. Student’s *t*-test was used to identify significant differences between treatment means at *p* < 0.05. Proteins with content that differed by ≥1.2-fold were considered differentially abundant proteins (DAPs).

### 2.7. Bioinformatics Analysis

The GO (http://geneontology.org, accessed on 22 October 2021) and KEGG pathway (https://www.genome.jp/kegg/, accessed on 22 October 2021) databases were used to perform GO function annotations and metabolic pathway analysis, respectively, on the DAPs to obtain information regarding their associated biological functions and processes and their cellular locations.

### 2.8. Western Blotting

Two key proteins were selected as targets to corroborate the results of the proteomics analyses and verify photosynthetic protein expression. The extracted *P. dentata* proteins were quantitated by SDS-PAGE, and the protein strips were transferred by a semi-dry method [23]. At a constant 400 mA, 45 min was required to transfer the target protein to the activated polyvinylidene fluoride (PVDF) membrane. The membrane was blocked with fast blocking solution for 10 min, primary rabbit anti-photosynthesis protein serum (1:3000) was added, and the membrane was incubated at 4 °C overnight. The secondary antibody was horseradish peroxidase (HRP)-labeled goat anti-rabbit serum (1:3000). The mixture was incubated for 2 h, and the membrane was immersed in western blot HRP-DAB substrate color developing solution, incubated in the dark for 5 min, and photographed with an automatic gel imager (3500R, Tanon, Shanghai, China).

## 3. Results

### 3.1. Identified by Label-Free Proteomic Analysis

**Figure 1:**
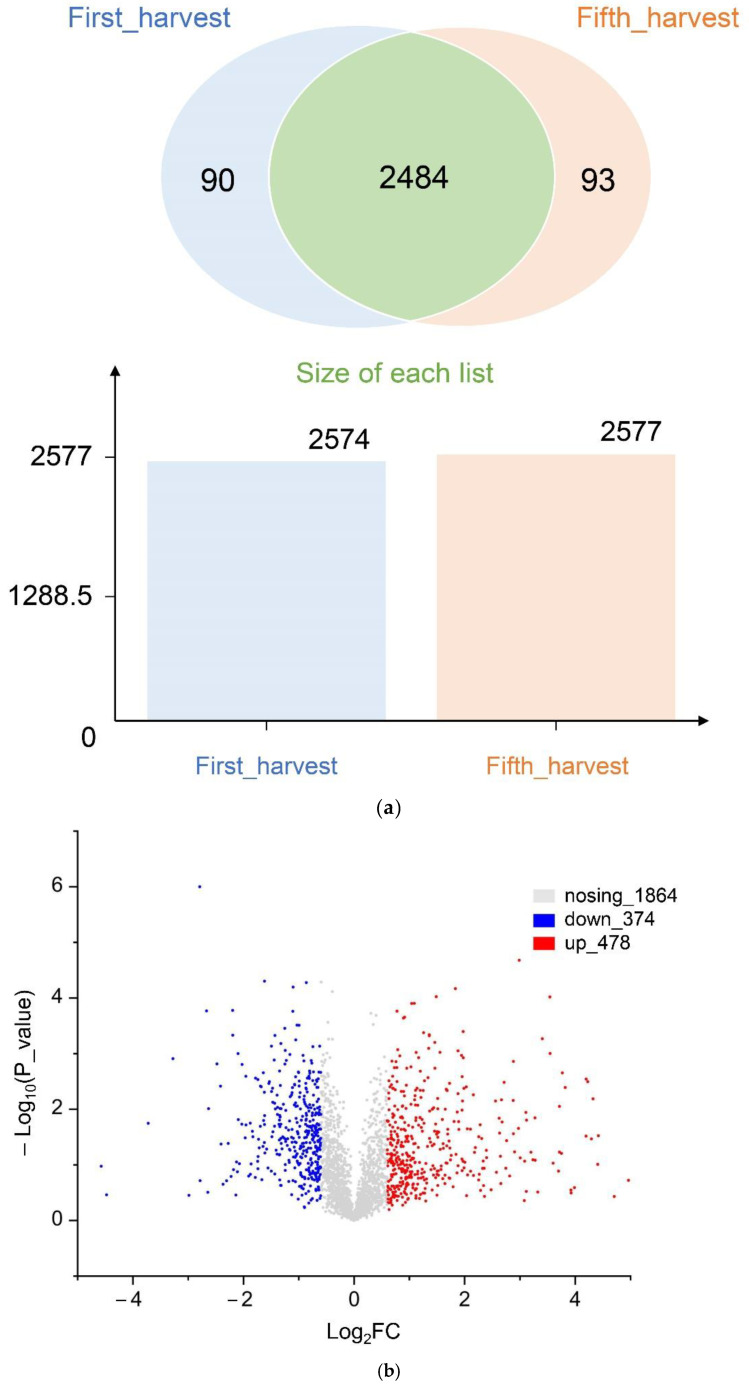
Label-free proteomic analysis: (**a**) First/Fifth harvest protein Venn of *P. dentata*. (**b**) Volcano plot of differentially abundant proteins (DAPs) from *P. dentata* (threshold = | log2 (fold change) |>1.2 or <0.83 and *p* < 0.05).

We identified a total of 5151 proteins in *P. dentata*. There were 2574 proteins at the first harvest, of which 90 were uniquely expressed. There were 2577 proteins at the fifth harvest, of which 93 were uniquely expressed (Figure 1a). Student’s *t*-test analysis identified 2716 DAPs, of which 478 were upregulated and 374 were downregulated. The criteria were as follows: FC > 1.2 indicated an upregulated protein while FC < 0.83 indicated a downregulated protein (*p* < 0.05) (Figure 1b).

### 3.2. GO and KEGG Pathway Annotation Analysis of the DAPs

**Figure 2:**
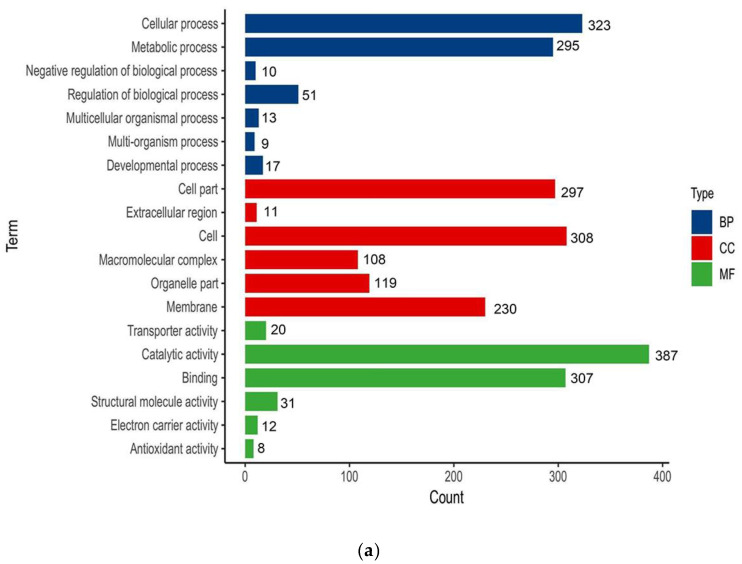

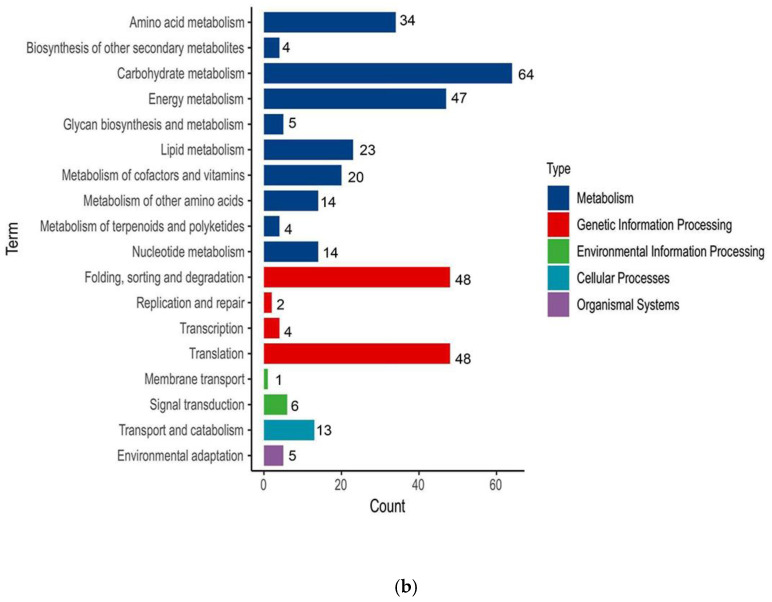
Annotation analysis of the DAPs: (**a**) Protein GO analysis of First/Fifth harvest (MF: Molecular function; CC: Cellular component; BP: Biological process). (**b**) Protein KEGG analysis of First/Fifth harvest.

The GO classification of the ADPs between the first and fifth harvest *P. dentata* groups is shown in Figure 2a. The annotations of the DAPs in biological processes were significantly increased and focused on cellular processes (GO: 0009987, 323), metabolic processes (GO: 0008152, 295), and regulation of biological processes (GO: 00050789, 51). In cell composition, cell (GO: 0005623, 308) and cell part (GO: 00044464, 297) were enriched. There were no significant differences in the cell membrane, membrane transport, or organelle expression. Under molecular functions, catalytic activity (GO: 00003824, 387), binding (GO: 0005488, 307), and other subsidiary proteins were enriched. The activation energy of the proteome continuously regulated *P. dentata* growth and development with the extension of the harvest period. The KEGG pathway annotation showed that metabolic proteins occupied the highest proportion, followed by genetic information processing and environmental information processing (Figure 2b). Under metabolic proteins, amino acid metabolism (34), carbohydrate metabolism (64), and energy metabolism (47) were enriched. These were presumed to be related to photosynthesis and respiration during *P. dentata* growth and development. The carbohydrates were converted into nucleic acids, proteins, lipids, and structural polysaccharides and contributed to the differences in algal quality among harvest periods [25]. Under genetic information, the folding, classification, and degradation (48) and translation (48) annotations were enriched. Under environmental information processing, guide protein signal transduction was presumed to be influenced by seawater and the ambient environment and gradually adapted to growth regulation.

### 3.3. GO and KEGG Pathway Enrichment Analysis of the DAPs

**Figure 3:**
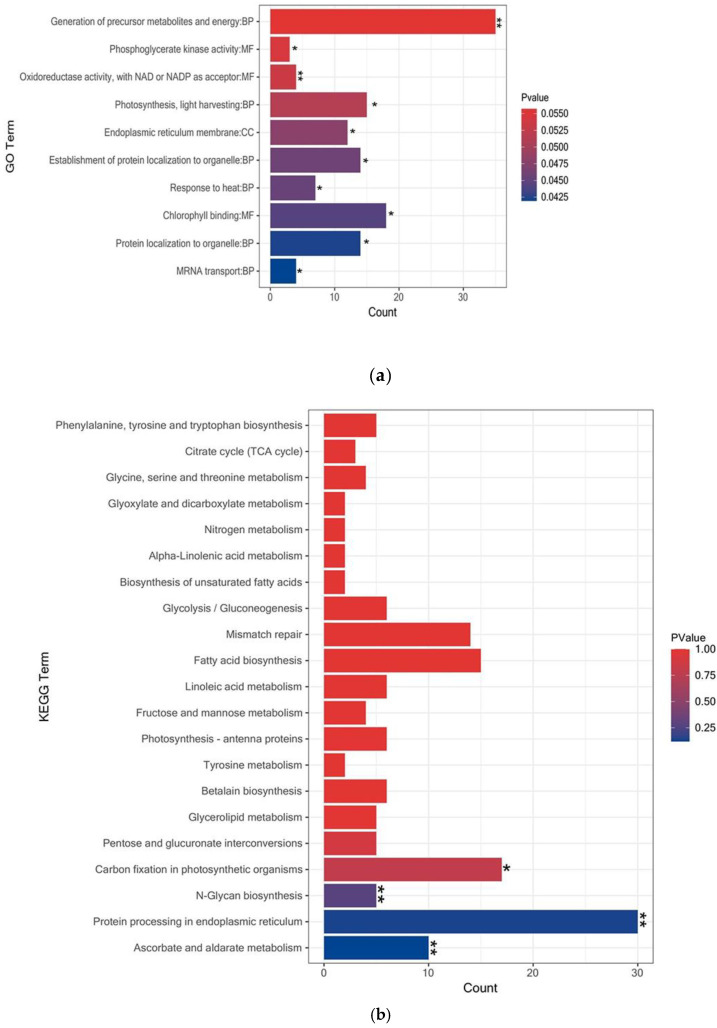
Enrichment analysis of the DAPs DAPs: (**a**) GO function of enrichment analysis of First/Fifth harvest. (**b**) DAPs KEGG function of enrichment analysis of First/Fifth harvest. (* *p* < 0.05, ** *p* < 0.01).

The molecular characteristics of the *P. dentata* proteins were established. A GO enrichment analysis was performed using goatools (https://pypi.org/project/goatools, accessed on 22 October 2021), and the molecular functions were elucidated and taxonomically categorized by Fisher’s exact test. The GO functional enrichment annotations found by the enrichment analysis are presented in Figure 3a. Photosynthesis, light harvesting (GO: 0007965), and organellar protein localization (GO: 0072594) had similar significance levels in the distribution. The activity levels of oxidoreductases using other nitrogenous compounds as donors and NAD or NADP as acceptors (Oxidoreductase activity, with NAD or NADP as acceptor, GO: 0016717) and metabolite precursor and energy generation (GO: 0006091) were the most highly enriched and significantly differently expressed (*p* < 0.01). The changes in respiratory metabolism and self-regulation influenced by exogenous sources were enhanced during *P. dentata* growth. During its growth, *Porphyra* is sensitive to environmental conditions (current, salinity, tides, pH, light intensity, and nutrient levels), and these contribute to variations in growth and quality. At the single-cell level, multicellular fertilization, attachment, germination, thallus development, and damage to the light-trapping pigment system and the algal body in response to external stressors all affect metabolite production and light energy transmission and, by extension, the levels of ATP and NADP required to reduce CO_2_ for carbohydrate biosynthesis. For these reasons, the two proteins classes significantly differed among *P. dentata* harvest times [26,27]. Based on the GO analysis, a KEGG pathway enrichment annotation was also performed. *p* < 0.05 was the correction threshold (Figure 3b). Proteins were significantly upregulated in the following pathways: protein processing in the endoplasmic reticulum (map04141), ascorbate and aldarate metabolism (map00053), N-glycan biosynthesis (map00510), and carbon fixation in photosynthetic organisms (map00710). Protein enrichment was also detected in betalain biosynthesis (map00965), linoleic acid metabolism (map00591), photosynthesis-antenna proteins (map00196), and others associated with nutrient biosynthesis.

### 3.4. Differential Expression of Photosynthesis-Related Proteins

**Figure 4:**
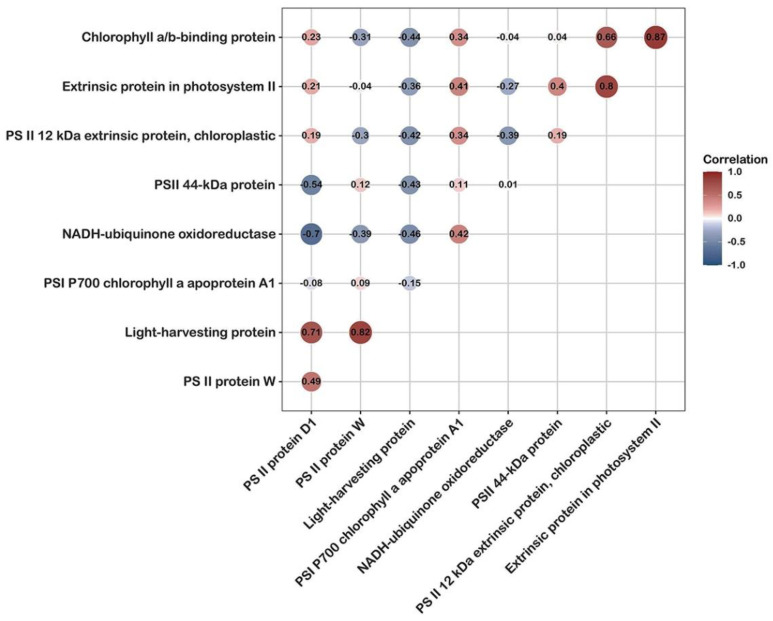
Correlation changes of important photosynthetic differential protein.

Photosynthesis is essential for algal growth and energy production. Half the products of CO_2_ fixation in the global ecosystem are derived from algal photosynthesis [28]. *P. dentata* is the main alga in the intertidal zone, and its quality may change during growth until harvest in response to changes in light intensity. Weather conditions, changes in the angle of light inclination, and other exogenous factors account for observed differences in the perceived stress responses manifested as protein upregulation, downregulation, and new protein emergence. A portion of the protein decomposes and ultimately disappears. Here, we selected nine DAPs associated with photosynthesis and calculated the correlations among them (Figure 4, Table 1).

In the photosynthetic system of *P. dentata*, the DAPs were distributed mainly in the chloroplasts and thylakoid membranes. The levels of PSI P700 chlorophyll a apoprotein A1, PSII protein D1, PSII 44kDa protein, PSII protein W, and chlorophyll a/b-binding protein were upregulated while the light-harvesting protein was downregulated at the first/fifth harvest. Extrinsic protein in PSII, NADH-ubiquinone oxidoreductase, and PSII 12 kDa extrinsic chloroplastic protein was specifically expressed at the fifth harvest.

### 3.5. Western Blotting

**Figure 5:**
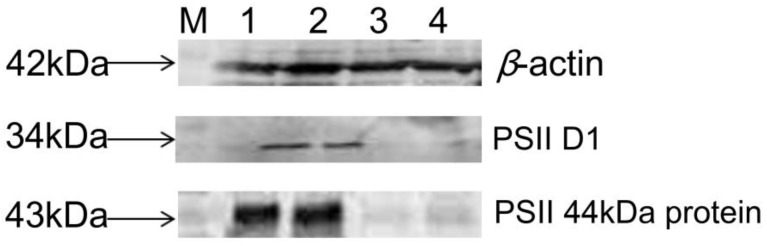
Differential proteins D1 and 44k Da verification result of western blotting (M: Marker 1~2: Fifth, 3~4: First).

When using β-actin as the internal reference, the changes in the expression level of certain proteins at the first/fifth harvest were verified by western blotting (Figure 5). Two parallel experiments were conducted, and the DAPs were D1 and 44-kDa in PSII. The target DAPs were upregulated at the first/fifth harvest. Hence, the western blotting results were in accordance with those of the proteomic assay.

## 4. Discussion

In algae, aerobic photosynthesis occurs mainly in Photosystems I and II (PSI and PSII), which dynamically absorb and balance light energy. The PSI and PSII comprise chloroplasts, light-harvesting antenna proteins, redox proteins, and other elements. It actively distributes the absorbed light energy for photosynthesis [29].

Photosystem II protein W (PSII W) belongs to the Psb28 protein family. It protects PSII catalyzing light-induced water oxidation, converting light energy into chemical energy, and generating O_2_. During assembly, Psb28 prevents PSII from being damaged by light. Gradual PSII W upregulation may occur as a result of a prolonged harvest period and gradual darkening of the algae. The latter potential confirmed increases in the efficiency of photosynthesis and the biosynthesis of light-harvesting pigment proteins. Excessive light energy absorption increases the synthesis of photoprotective PSII W, thereby ensuring normal algal growth and development. The lack of this protein may result in growth retardation, especially under naturally occurring intermittent high light/dark conditions. The regulation of this protein confirms the importance of the PSII assembly factors in light adaptability [30,31].

Factors such as high-intensity light and high temperature reduce CO_2_ availability, induce reactive oxygen species (ROS) formation, and significantly reduce the photosynthetic rate of *Porphyra* chloroplasts [32]. Protective extrinsic proteins are attached to the periphery of PSII. The PSII 12-kDa extrinsic protein, which is present in the chloroplast and is a member of the Psbu, is an extrinsic protein in PSII of cyanobacteria and red algal and was specifically expressed at the fifth harvest. It stabilizes the photosynthetic environment in the presence of oxygen [33]. It is possible that prolonged photosynthesis stimulates photoprotection, mitigating the adverse impact of external abiotic stress. In addition, the extrinsic protein in PSII reduces harvest loss that is caused by high ROS levels. This is a self-defense mechanism in algae [34,35]. NADH ubiquinone oxidoreductase is an energy-transducing, ATP-synthesizing enzyme that participates in the PSI electron transport chain (ETC) during light energy conversion. This enzyme is specifically expressed during the fifth harvest as PSII activity is inhibited from balancing the ETC and normal ATP synthesis. NADH ubiquinone oxidoreductase maintains normal ATP cycle activity while inhibiting excessive substrate reduction and ensuring normal biosynthesis [36,37].

In *P. dentata*, PSII D1 is an important protein in light energy conversion. The synthesis and degradation of PSII D1 play decisive roles in algal quality and recovery. When the light intensity exceeds the energy synthesis requirement, it can readily cause mechanical damage to the PSII system and trigger photoinhibition [38]. Consequently, the pigment content of the laver thallus decreases as the synthesis of carotenoids, phycoerythrins, phycocyanins, and other pigments is reduced. The algal thalli may undergo discoloration, decay, and deterioration [39]. Chloroplast thylakoid membrane fluidity is the key to damaged D1 protein degradation and synthesis [40]. The western blotting and proteomic analyses showed that the D1 protein content was significantly higher at the fifth than the first harvest. As the harvest period extended, the laver thalli gradually became wider, thicker, and darker. The amount of light energy absorbed by the photosynthetic pigment protein was reduced, but the relative increase in energy conversion efficiency nonetheless maintained the rate of photosynthetic oxidation. The thick algal thalli mitigated light-induced damage and promoted D1 protein synthesis. There were higher levels of Na+-ATPase at the fifth than the first harvest, and high ATP conversion efficiency was maintained. The results of a prior study on the photosynthetic characteristics of *Porphyra yezoensis* at different harvesting periods were similar to those of the present work [41].

Photosynthesis is affected by the carbon cycle and UV irradiation. The latter inhibits algal carotenoid and chlorophyll biosynthesis, especially in dehydrated *Porphyra*. Thence, the loss of photosynthetic pigment significantly lowers photochemical efficiency and photosynthetic carbon fixation by PSII. However, gradual increases in atmospheric CO_2_ and UV adaptability help improve *P. dentata* growth and development. These modifications induce photosynthetic activity and repress photoinhibition. Therefore, prolongation of the algal harvest period significantly increases the D1 protein content. Rubisco is the key protein in carbon fixation and was upregulated in the first/fifth algal harvests. Under natural conditions, UV irradiation is conducive to the formation and accumulation of amino acids required for protein synthesis. Additionally, mycosporine-like amino acid (MAA) proteins unique to *Porphyra* increase throughout the harvest period and mitigate the effects of UV radiation and oxidative stress, promoting PSII D1 protein synthesis [42]. Previous research on *Porphyra haitanensis* reported that UV irradiation significantly increased the proline (Pro), serine (Ser), histidine (His), and alanine (Ala) content which, in turn, could offset stress-related cell damage, remove ROS, protect membrane integrity, enhance metabolic pathways, and increase the total sugar content [43,44]. These findings were consistent with our results (data not published). Glutamic acid (Glu) was downregulated in *Porphyra haitanensis* but upregulated in *P. dentata,* possibly because each species has a unique background antioxidant capacity. An increase in the glutamate content can stimulate glutathione metabolism and increase the ascorbate content. Light stress induces the production of dehydroascorbic acid (DHA), which reduces oxidative damage. Increases in amino acid content enhance the high-quality protein content of algal supplements. Moreover, the fifth algal harvest has a strong seaweed flavor and can be used as high-quality raw material for seaweed processing.

The exogenous ion content in *P. dentata* significantly affects its photosynthetic rate. The Ca^2+^ and Fe^3+^ content significantly increased in *P. dentata* with prolongation of the harvest period (data not published). Increases in the concentrations of these cations maintain cellular homeostasis and redox capacity, mitigate light-induced temperature elevation, improve antioxidant capacity, and favor D1 protein synthesis [45]. Mn^2+^ is also a key cofactor in the latter process. The C-terminus of mature D1 protein is critical for Mn^2+^ cluster assembly. The affinity of the PSII external proteins is significantly reduced in the absence of Mn^2+^ and its cluster elements. The loss of D1 protein hinders further biosynthesis of this substance. Free Ca^2+^ is also necessary for D1 protein assembly. For this reason, the Mn^2+^ content was significantly higher at the fifth than the first algal harvest [46].

The 44-kDa CP43 antenna protein in PSII is the core component of the antenna system and is vital to light energy absorption and transmission, and pigment biosynthesis. CP43 transfers light energy to the PSII reaction center (RC) and help protect the optical system structure. The PSII D1 and PSII 44-kDa protein levels are mutually correlated. D1 maturation is severely impeded when photoinhibition alters the PSII steady-state. Hence, the interaction between D1 and CP43 is hindered, as our western blotting results demonstrated [47]. However, the CP43 and D1 proteins accumulate and repair, the pigment proteins are synthesized and accumulate, and the algal color deepens with the gradual extension of the growth and harvest periods. In addition, CP43 forms a complex domain with the surrounding CP47 protein. This mechanism is conducive to light energy storage, increases the content of pigment proteins such as cytochrome b6, and accelerates color accumulation, as our measurements indicated [48].

Members of the Lhc pigment protein family are differentially expressed in *P. dentata*. They all capture light energy and rapidly transmit energy. Lhc is regulated by the photochemical reactions in PSI and PSII [49]. In *P. dentata*, Lhc localized mainly to PSI and various Lhch1 species. The latter are light-harvesting antenna pigment protein complexes that participate in chlorophyll biosynthesis and color assembly in *P. dentata*. Lhca1 protein forms an LHCI-730 heterodimer when Lhca4 and PSI core proteins combine with PsaG and PsaF after delivery to PSII. They function in tandem with PSII D1, PSII D2, CP43, and CP47 proteins to catalyze NADP synthesis [50]. Light-harvesting protein content gradually decreased, and chlorophyll a/b-binding protein gradually increased at the first/fifth harvests. In the initial growth stage, a large amount of light must be absorbed to capture sufficient light energy to support the energy response and ATP synthesis and conduction. Later in the season, reactions decline in the efficiency of photosynthesis induces algal senescence and pigment accumulation [51]. Potential description the quality of *P. dentata* was lower at the fifth than the first harvest.

## 5. Conclusions

In the present study, a label-free proteomics approach was used to investigate the changes in photosynthetic protein content of *P. dentata* harvested at different times. A total of 2716 DAPs were identified. Of these, nine major photosynthetic DAPs were screened and confirmed. Western blotting verified that the PSII D1 and 44-kDa protein levels gradually increased throughout the harvest period. The results of this study showed that the expression levels of specific proteins protecting the photosystem increased with harvest time, accelerating algal growth and pigment accumulation, gradually causing algal hypertrophy, reducing organoleptic quality, increasing carbohydrate accumulation, lowering the total protein content, and diminishing overall edible quality. The findings of this work emphasize the importance of photosynthetic protein changes during the harvest season, which sheds light on the molecular mechanism of nutritional changes in *P. dentata* during this period. At the same time, a protein database was constructed to support the future quality classification of this commercially valuable red algae. This lays a theoretical foundation for research on the breeding, cultivation, and processing of *P. dentata*, and provides an important reference for better understanding the physiological response mechanisms of marine algae to natural environmental stresses.

## Figures and Tables

**Table 1:** Differentially abundance proteins for photosynthesis in First/Fifth harvest identified by label-free proteomics.

Accession	Protein Name	Ko Name	First/Fifth Harvest FC	*p*-Value
TRINITY_DN4391_c0_g1_i1_orfp1	Extrinsic protein in photosystem II	Xdh	129.68	0
TRINITY_DN20824_c0_g2_i1_orf1	Photosystem I P700 chlorophyll a apoprotein A1	PsaA	1.625	0.012
TRINITY_DN43883_c0_g1_i1_orf1	Photosystem II protein D1	PsbA	1.363	0.031
TRINITY_DN8817_c0_g1_i1_orf1	Photosystem II 44 kDa protein	psbC	1.485	0.020
TRINITY_DN14993_c0_g1_i1_orf1	Photosystem II protein W	Psb28	1.235	0.093
TRINITY_DN12291_c0_g1_i1_orfp1	Photosystem II 12 kDa extrinsic protein, chloroplastic	PsbU	129.68	0
TRINITY_DN376_c0_g2_i1_orfp1	NADH-ubiquinone oxidoreductase		129.68	0
TRINITY_DN31442_c0_g1_i1_orf1	Chlorophyll a/b-binding protein	Lhca1	1.891	0.321
TRINITY_DN2334_c0_g3_i3_orf1	Light-harvesting protein	Lhca1	0.762	0.142

## Data Availability

The original contributions presented in the study are included in the article, further inquiries can be directed to the corresponding authors.
